# Personal history of irradiation and risk of breast cancer: A Mendelian randomisation study

**DOI:** 10.7189/jogh.14.04106

**Published:** 2024-10-11

**Authors:** Yaoyao Liu, Zeyu Liu, Jiaru Chen, Manfeng Liang, Chunqing Cai, Fei Zou, Xueqiong Zhou

**Affiliations:** Department of Occupational Health and Medicine, Guangdong Provincial Key Laboratory of Tropical Disease Research, School of Public Health, Southern Medical University, Guangzhou, China

## Abstract

**Background:**

Studies on the relationship between personal history of irradiation and breast cancer have been reported for a long time. Still, epidemiological studies have not been conclusive, and the causal relationship is unclear. To address this issue, we employed Mendelian randomisation (MR) analysis to examine the association between individual radiation exposure history and breast cancer.

**Methods:**

We used a series of quality control methods to select single nucleotide polymorphism (SNP) closely related to exposure. Meanwhile, several analysis methods were used to analyse the sample data to make the conclusion more reliable. To evaluate the horizontal pleiotropy, heterogeneity and stability of SNPs for breast cancer, the MR-Egger intercept test, Cochran’s Q test and ‘leave one’ sensitivity analysis were used. Finally, the outlier variation determined by the Mendelian Randomisation Pleiotropy RESidual Sum and Outlier test is gradually eliminated to reduce the influence of heterogeneity and horizontal pleiotropy.

**Results:**

After implementing rigorous quality control procedures, we carefully chose 102 qualified instrumental variables closely associated with the selected exposure for sensitivity analysis. This was conducted to evaluate the heterogeneity, level multiplicity, and stability of SNPs in the context of personal radiation history and its correlation with breast cancer. The results of the inverse variance weighted method analysis revealed a positive correlation between personal radiation and a heightened risk of breast cancer (odds ratio (OR) = 1.52; 95% confidence interval (CI) = 1.30–1.77). We also validated on another data set; the results were similar (OR = 1.51; 95% CI = 1.27–1.81). Furthermore, the findings from the sensitivity analysis were consistent. At the genetic level, our research demonstrated that personal radiation exposure is associated with an elevated risk of breast cancer.

**Conclusions:**

Using genetic data provides evidence and strengthens the causal link that personal radiation causes breast cancer.

Breast cancer boasts the highest incidence rate and has become a major focal point in the realm of global public health [1–3]. It primarily affects women, posing a significant threat to their health, and it accounts for the highest proportion of female-related diseases [4,5]. Breast cancer exhibits rapid growth, a propensity for metastasis, a high likelihood of recurrence following treatment, and often develops resistance to drugs, leading to suboptimal treatment outcomes [6]. Approximately 3–10% of breast cancer patients present with distant metastasis at the time of diagnosis, with the majority progressing to advanced-stage cancer. Concurrently, the recurrence rate of breast cancer is on the rise [7,8]. Breast cancer is an aggressively invasive tumour, and while chemotherapy remains the primary treatment modality, its effectiveness remains constrained [9].

Breast cancer arises from a combination of environmental and genetic factors. Epidemiological and laboratory studies indicate that environmental determinants encompass organic solvents and ionising radiation, while genetic elements involve age, family history, and reproductive aspects [10–12]. Given the omnipresence of radiation in everyday life, including sources such as computers, mobile phones, industrial processes, and medical diagnostics, the influence of radiation on breast cancer assumes significant importance. The breast is particularly susceptible to radiation-induced cancer [13]. The association between radiation and breast cancer has been the subject of extensive research. A significant portion of the data regarding this connection is derived from studies involving atomic bomb survivors and women exposed to radiation for diagnostic or therapeutic reasons [14]. Previous research on the relationship between radiation and breast cancer has primarily centred on atomic bombs and nuclear accidents, yet the findings have not been consistent across all studies. While some investigations have revealed a positive correlation between breast cancer and radiation [15,16], others have found no significant association [17,18]. Solar UV radiation can decrease the occurrence of breast cancer, but this effect is specific to certain types of breast cancer [19,20]. Radiation exposure during infancy and undergoing a computerised tomography scan for medical reasons elevate the risk of developing breast cancer [21–23]. The tumorigenicity of radiation is uncertain and influenced by numerous factors. Different populations exhibit varying radiation tolerance levels, with notable racial and ethnic disparities, alongside environmental influences [24,25].

Nonetheless, because of the presence of confounding factors in epidemiological observational studies and the absence of data from randomised controlled trials within this context, it remains uncertain whether these associations can be definitively attributed to causality. To bolster the connection between individual exposure to radiation and breast cancer and substantiate its causal relationship, we undertook a Mendelian randomisation study. Utilising genetic variation as an instrumental variable for exposure (such as personal radiation) through the Mendelian randomisation (MR) design can significantly fortify causal inferences. This methodology effectively mitigates the influence of confounding variables. Given that genetic variation at the gene level is randomly allocated during pregnancy, it typically exhibits no association with potential confounding factors, including environmental elements. In the context of MR design, matching effector alleles mirrors the randomisation process in controlled trials. Furthermore, this approach allows for the direct establishment of a causal relationship between the two variables, as the genetic variants used to substitute exposure effects remain unaffected by the occurrence and progression of outcomes [26]. MR study was conducted to ascertain the potential causal link between individual radiation exposure and the risk of breast cancer.

## METHODS

### Study design

**Figure 1:**
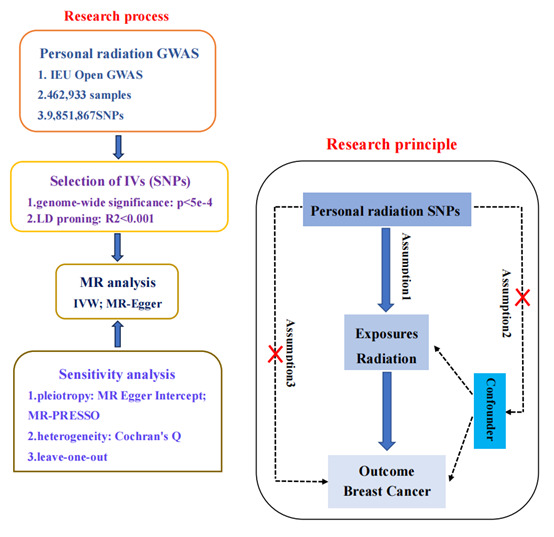
Study design flowchart. GWAS – genome-wide association study, SNP – single nucleotide polymorphism.

MR analysis is a statistical method that employs genetic variation as an instrumental variable [27]. There are three important assumptions in MR analysis. The first hypothesis posits that genetic variation should be strongly associated with exposure outcomes when employed as an instrumental variable. The second hypothesis proposes that the chosen genetic variants must be independent and devoid of any connections to confounding factors. The third hypothesis stipulates that the selected gene variants should exclusively influence the outcome through risk factors [26]. This study relies on publicly accessible data from genome-wide association studies (GWAS) conducted at the general population level. **Figure 1** outlines the design process.

### Genetic instrument selection

The data set of GWAS provides reliable data sources and instrumental variables for MR analysis in this study. This study’s aggregate statistical data for cancer were sourced from the Integrative Epidemiology Unit Open GWAS, comprising 462 933 and 337 159 samples, respectively. The sample in this study was derived from the population of inpatient cases in the UK biological sample. Since fewer single nucleotide polymorphisms (SNPs) meet the stringent threshold (*P* < 5e −8), we have opted to use a more lenient threshold (ranging from *P* < 5e −8 to *P* < 5e −4) for SNP selection [28]. The selected SNPs were identified using the ‘clump’ parameter set to ‘true’, which allowed for removing linkage disequilibrium. The parameters ‘r^2^’ (r^2^ is a value between zero and one, where r^2^ = 1 indicates complete linkage disequilibrium between two SNPs, while r^2^ = 0 signifies complete linkage equilibrium, meaning their assignment is completely random) and ‘kilobase’ was configured with values of 0.001 and 10 000, respectively. All SNPs and related data were sourced from studies exclusively analysing populations of European ancestry to mitigate potential biases stemming from demographic stratification.

### Statistical analysis

The inverse variance weighted method (IVW), serving as the primary statistical model for MR, encompasses two variants – random effects IVW and fixed effects IVW. A random effects model is selected in cases of pronounced heterogeneity (*P* < 0.05). Conversely, both models can be employed when heterogeneity is not pronounced [29]. The random-effects IVW method was employed to assess the correlation between radiation and breast cancer at the gene level. For the analysis of multiple SNPs, the IVW fixed-effect method was utilised to evaluate the association between radiation and breast cancer at the gene level. Causal estimates were derived through meta-analysis of SNP-specific Wald ratio estimates, which involve the beta coefficient for the SNP’s effect on the outcome divided by the beta coefficient for the SNP’s effect on the exposure. This meta-analysis employed either a random-effects or fixed-effects inverse variance method, which assigns weights to each ratio based on its standard error [27]. The standard error of the ratio estimate is estimated using the delta method. We employed the IVW method as our primary analytical approach. IVW utilises the inverse variance method to assign weights to the association between SNPs and outcomes, yielding a consolidated causal estimate for each SNP. This method is renowned for its robust statistical power, making it an effective tool for estimating the causal relationship between exposure and outcomes [30].

Three sensitivity analyses were performed, including the weighted media, MR-Egger and Mendelian Randomisation Pleiotropy RESidual Sum and Outlier (MR-PRESSO) approaches [31–33]. Theoretically, if 50% of SNPs are valid, the weighted median method can produce a consistent causal estimate [31]. When SNPs related to exposure indirectly influence the outcomes through pathways other than the assumed direct exposure, it leads to horizontal pleiotropy. MR-Egger regression can detect and correct the possible horizontal pleiotropy, and when the MR-Egger intercept *P*-value of the intercept >0.05 indicates no horizontal pleiotropy [32,34,35]. MR-PRESSO can detect outliers in the data and make causal estimates after removing the outliers [33]. Considering there may be a large sample overlap between the exposure factor and the outcome database, the F-statistic (F) is calculated to measure the strength of the instrumental variables selected from the database and exclude the bias of sample overlap [36]. The radiation-related F-statistic exceeds 10, signifying that the causal association remains unaffected by weak instrumental variable bias, thus establishing the reliability of the study results. Cochrane Q value was used to assess the heterogeneity among estimates of SNPs in each analysis. All analyses were performed using two-sided tests with the MR-PRESSO package in R, version 4.2.3 (R Core Team, Vienna, Austria).

## RESULTS

**Figure 2:**
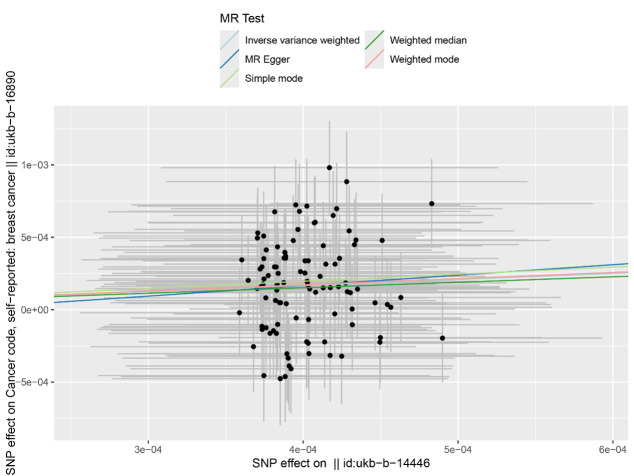
This scatter plot visually illustrates the causal effect of individual radiation on the risk of total breast cancer.

**Figure 3:**
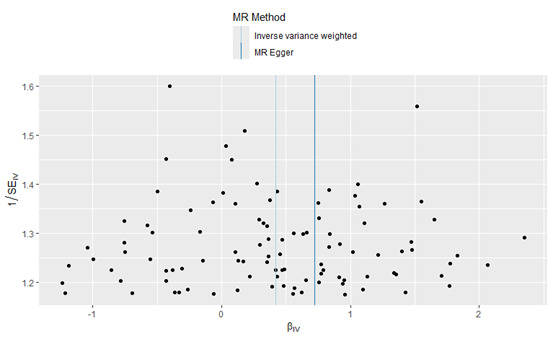
Funnel plot illustrating the causal relationship between individual radiation and total breast cancer.

**Figure 4:**
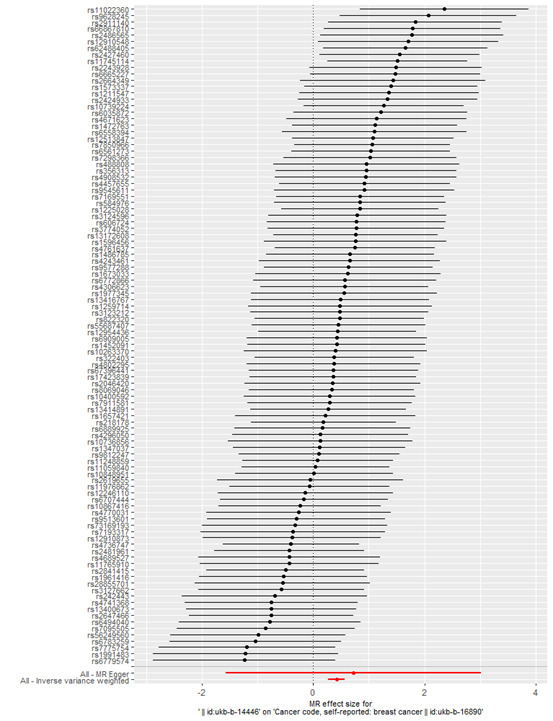
Forest plots depicting the impact of each SNP on the risk of total breast cancer. SNP – single nucleotide polymorphism.

**Figure 5:**
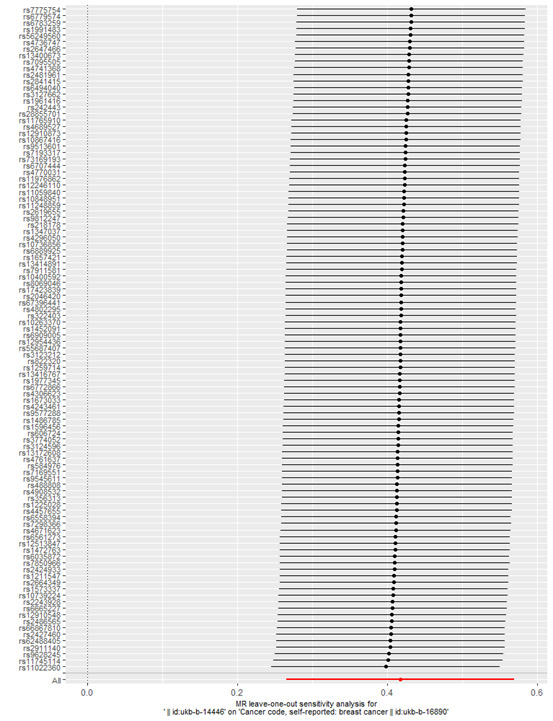
Assessing the causality of individual radiation exposure in relation to total breast cancer risk with each SNP excluded. SNP – single nucleotide polymorphism.

The IVW analysis revealed a causal relationship between individual radiation exposure and total breast cancer, with an odds ratio (OR) = 1.52; 95% confidence interval (CI) = 1.30–1.77, *P* = 7.869e–08 (**Table 1**). In the MR Egger analysis, the *P* > 0.05 indicates the absence of pleiotropy and ensures the results’ reliability. A causal scatter plot depicting individual radiation and the risk of total breast cancer illustrates that individual radiation is a significant risk factor for total breast cancer (**Figure 2**). The funnel plot (**Figure 3**) demonstrates that when a single SNP is utilised as an instrumental variable, the causal effect points are symmetrically distributed, suggesting minimal susceptibility to potential bias. **Figure 4** displays the individual effects of each SNP on total breast cancer. Sensitivity analysis revealed that there was no significant difference in the estimated results even when removing SNPs one by one. Therefore, it is evident that the estimated effect cannot be attributed to any single SNP (**Figure 5**). Similarly, we employed a different data set for validation and achieved consistent outcomes (Table S1 and Figures S1–4 in the **Online Supplementary Document**).

**Table 1:** Association of genetically predicted radiation with risk of breast cancer in sensitivity analyses

Method	SNP (n)	*P*-value	OR (95% CI)
MR-Egger	102	5.382e–01	2.06 (0.21–20.43)
Weighted median	102	1.247e–03	1.46 (1.16–1.84)
Inverse variance weighted	102	7.869e–08	1.52 (1.30–1.77)
Simple mode	102	1.359e–01	1.63 (0.86–3.10)
Weighted mode	102	1.996e–01	1.53 (0.80–2.94)

CI – confidence interval, OR – odds ratio, SNP – single nucleotide polymorphism

## DISCUSSION

Our findings indicate a positive correlation between individual exposure to radiation and the risk of breast cancer. This finding aligns with the majority of epidemiological studies on the relationship between radiation and breast cancer, although not all of them yield consistent results.

The inconsistent conclusions of previous epidemiological studies may be attributed to several factors, including variations in exposure dose, age at exposure, and individual differences [37,38]. Researchers have turned their attention to the cellular level, aiming to overcome the limitations posed by epidemiological population analysis. Different doses of radiation have varying effects on cells, and higher doses can lead to irreversible changes. For instance, exposure to four Gray units (Gy) of radiation can cause severe and permanent alterations [39]. Ionising radiation can induce the differentiation of non-breast cancer stem cells into a carcinogenic type [40]. Furthermore, studies have reported an increase in the invasiveness of surviving triple-negative breast cancer cells following exposure to ionising radiation [41]. In animal experiments, adult female rats were administered a single systemic dose of four Gy. Subsequently, an increased incidence of cancer was observed in the exposure group compared to the control group following radiation exposure [42].

Radiation is omnipresent in daily life, and due to lifestyle changes, we frequently encounter low-dose radiation in our everyday activities. This exposure poses a public health concern as it occurs in the general environment and occupational settings. The risk of breast cancer is associated with medium and high doses of radiation in atomic bomb survivors and radiotherapy patients. However, the effects of low-dose personal radiation on breast cancer risk remain unclear. Research suggests that even long-term exposure to low doses of radiation can contribute to the onset and progression of breast cancer [43]. Exposure to low-dose ionising radiation has been shown to enhance the invasiveness of breast cancer cells [44]. Research indicates that residents living in areas contaminated by radioactive dust from the Chornobyl accident may face an increased risk of breast cancer among women due to long-term exposure to low-dose radiation [45]. This aligns with our research findings, indicating a positive correlation between personal radiation exposure and overall breast cancer risk, thereby establishing it as a significant risk factor.

This study possesses both advantages and limitations. Its primary strength lies in the utilisation of MR analysis, which strengthens the causal inference between personal radiation and breast cancer. However, a limitation of this study is its focus on a specific population of European individuals, which introduces the possibility of population bias. Consequently, the generalisability of our findings to other populations might be limited. The samples of exposure and outcome data exhibit considerable overlap, posing a risk of overfitting the model and potentially inflating the observed correlations, thus affecting the accuracy of causal estimation [46]. However, F>10 suggests that the potential impact of sample overlap on the estimated deviation may be minimal.

## CONCLUSIONS

This study has successfully identified a positive correlation between individual radiation exposure and breast cancer risk at the genetic level. The findings not only provide genetic evidence that aligns with previous epidemiological studies but also explicitly establish the causal relationship between individual radiation exposure and breast cancer.

## Additional material


Online Supplementary Document
