# Supplementary material for: Personal history of irradiation and risk of breast cancer: A Mendelian randomisation study
Source: J Glob Health. 2024 Oct 11;14:04106. doi: 10.7189/jogh.14.04106 (PMC11467774; doi:10.7189/jogh.14.04106)
Supplement: Online Supplementary Document [file jogh-14-04106-s001.pdf]

Results related to another dataset (ukb-a-55)

Supplementary table 1. Association of Genetically Predicted Radiation With Risk of Breast Cancer in Sensitivity Analyses.

**Method**	**nsnp**	**p**	**or**	**or-lci95**	**or-uci95**
MR-Egger	102	7.598e-01	1.52	0.10	22.47
Weighted median	102	3.031e-02	1.33	1.03	1.72
Inverse variance weighted	102	5.728e-06	1.51	1.26	1.81
Simple mode	102	7.991e-01	1.10	0.54	2.22
Weiahted mode	102	8.520e-01	1.07	0.54	2.10

*nsnp-single nucleotide polymorphism's number. p-p-value. or-odds ratio.ci-confidence interval. lci95-lower ci95. uci95-upper ci95.

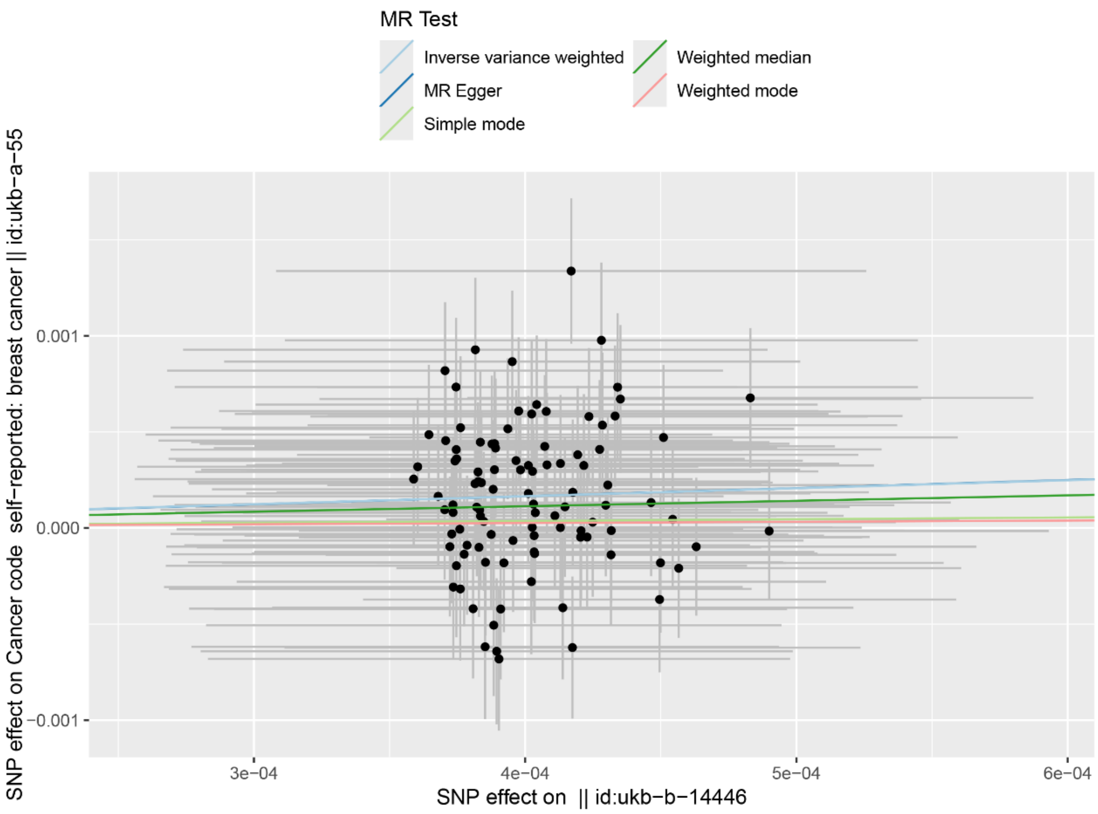

Supplementary figure 1. This scatter plot visually illustrates the causal effect of individual radiation on the risk of total breast cancer.

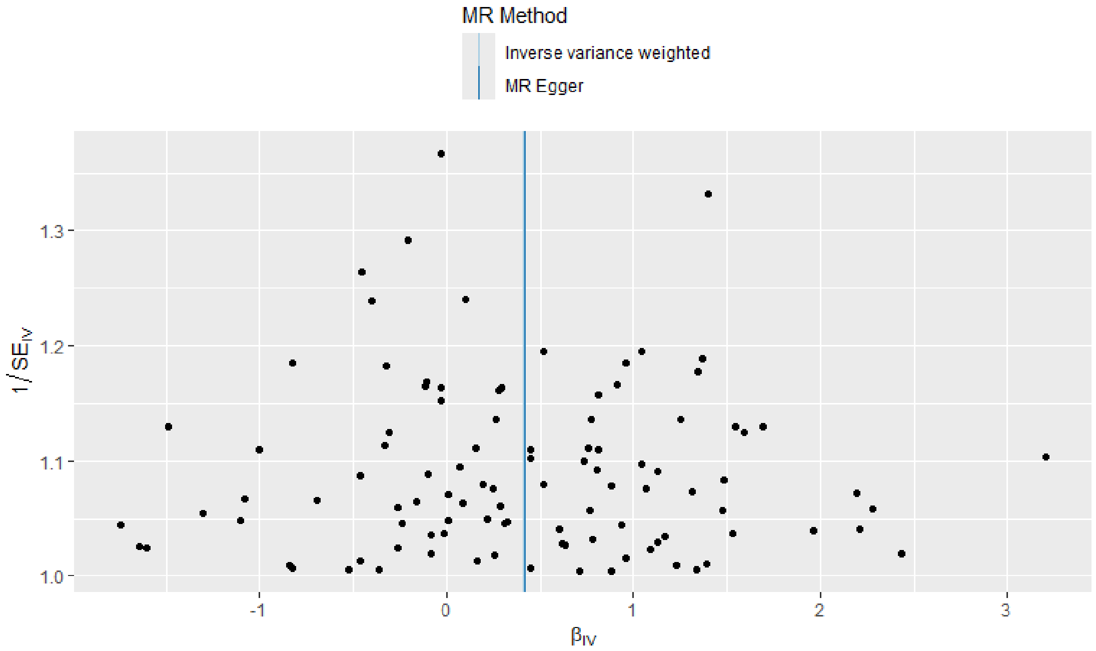

Supplementary figure 2. Funnel plot illustrating the causal relationship between individual radiation and total breast cancer.

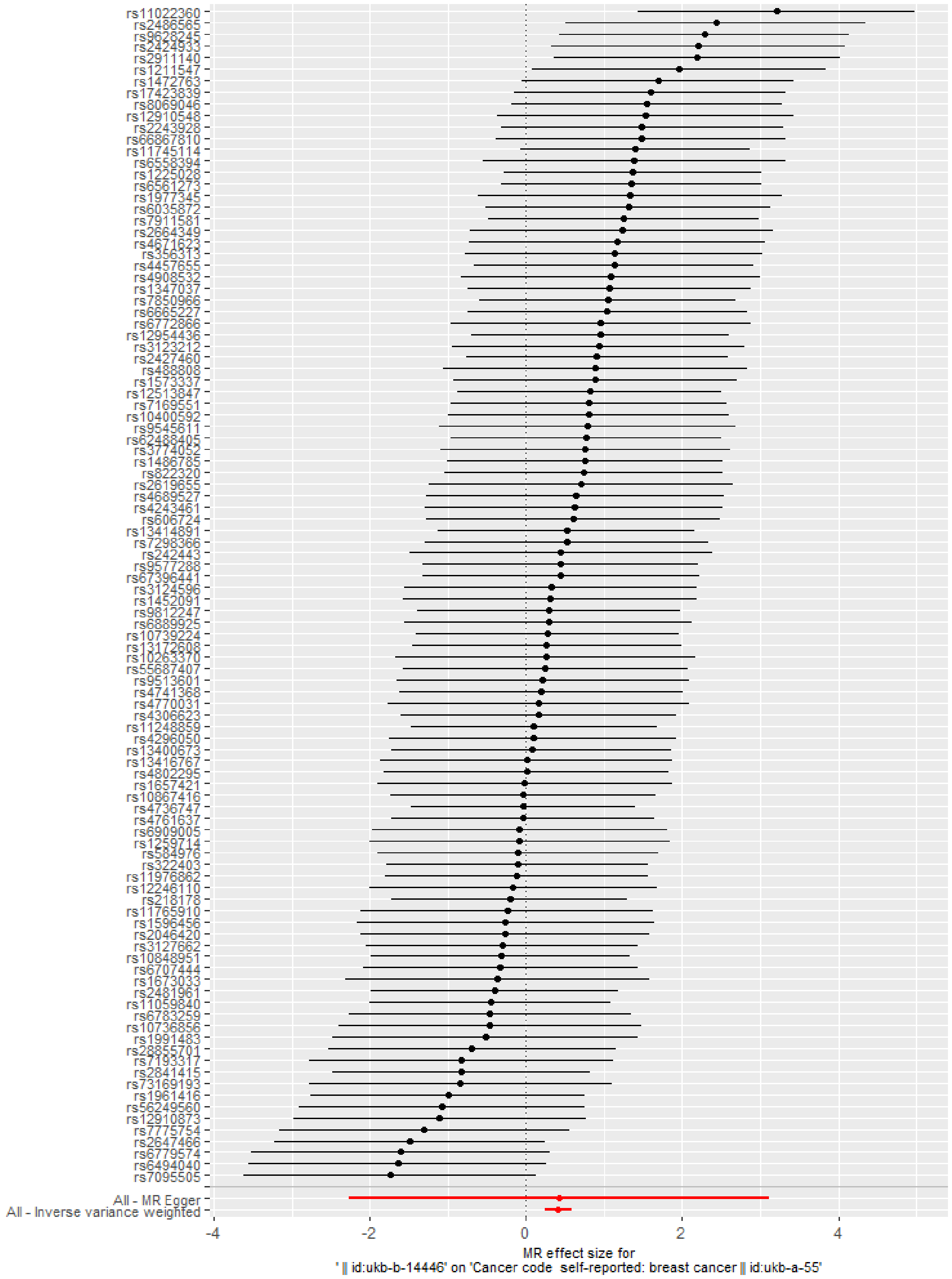

Supplementary figure 3. Forest plots depicting the impact of each SNP on the risk of total breast cancer.

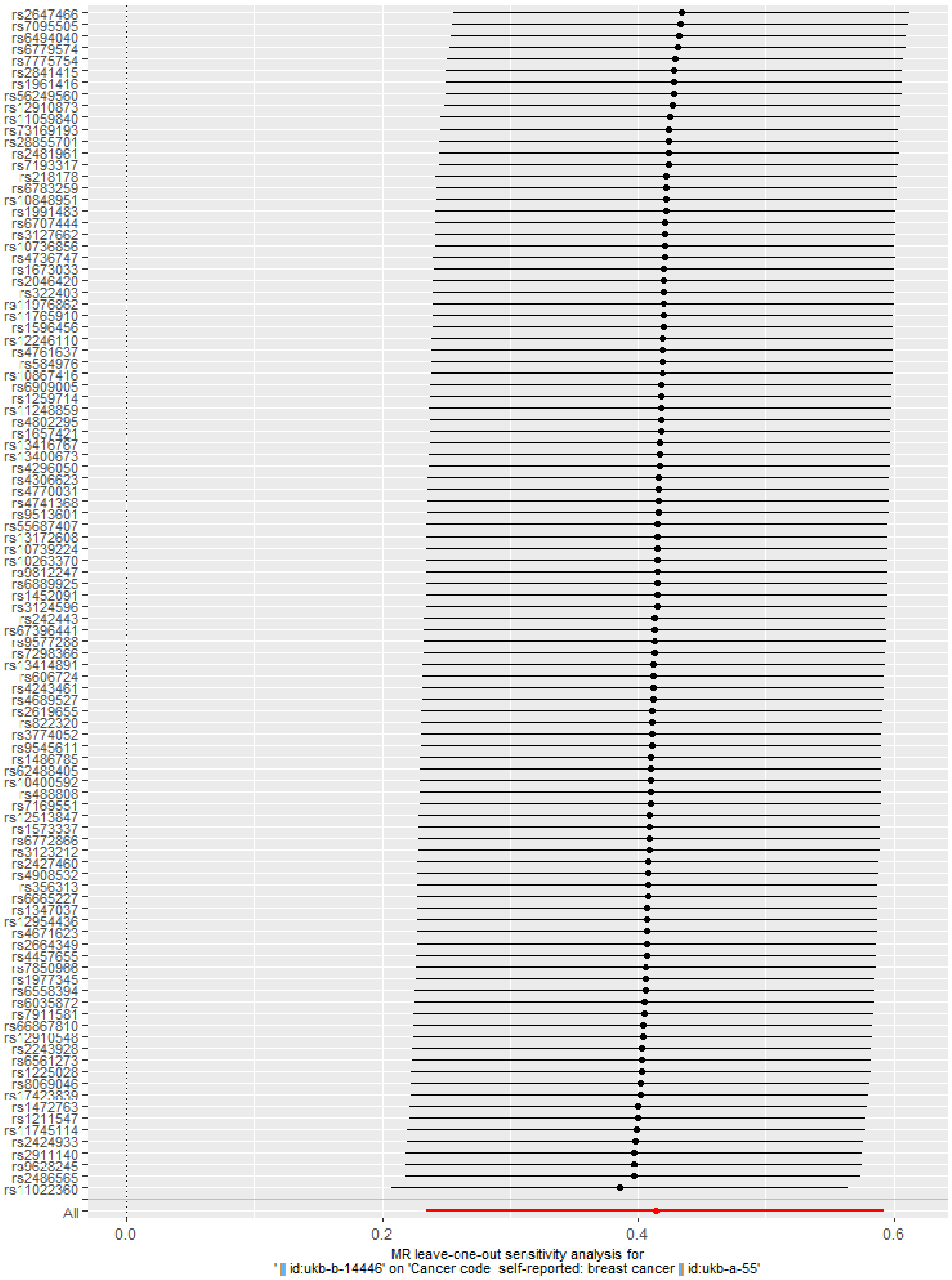

Supplementary figure 4. Assessing the causality of individual radiation exposure in relation to total breast cancer risk with each SNP excluded.

ukb-b-16890 SNPs

	SNP	effect allele.exposure	other allele.exposure	effect allele.outcome	other allele.outcome	beta.exposure	beta.outcome	eaf.exposure	eaf.outcome	chr	pos	se.outcon	samplesiz	pval.outc	samplesiz	se.exposure	pos.exposure	chr.expos	pval.exposure
1	rs10263370	T	C	T	C	-0.000370343	-0.000145988	0.462431	0.462427	7	75413948	0.0003	462933	0.64	463010	0.00010478	75413948	7	0.000409996
2	rs10400592	A	G	A	G	0.000408049	0.000120196	0.345315	0.345305	12	73131071	0.0003	462933	0.71	463010	0.00010769	73131071	12	0.00015
3	rs10736856	T	C	T	C	0.000385383	0.000048116	0.679632	0.679627	9	135490787	0.0003	462933	0.88	463010	0.00010969	135490787	9	0.000439997
4	rs10739224	G	T	G	T	0.000429701	0.00054438	0.376959	0.376965	9	109450946	0.0003	462933	0.085	463010	0.00010641	109450946	9	5.39995E-05
5	rs10747846	G	C	G	C	0.000389305	9.16133E-06	0.511668	0.511676	12	59928206	0.0003	462933	0.98	463010	0.00010263	59928206	12	0.00015
6	rs10848951	C	T	C	T	0.000431645	4.61126E-06	0.397641	0.397635	12	3996234	0.0003	462933	0.99	463010	0.00010517	3996234	12	4.09996E-05
7	rs10867416	C	T	C	T	-0.00043174	0.000103862	0.655657	0.655662	9	82324152	0.0003	462933	0.75	463010	0.0001079	82324152	9	6.29999E-05
8	rs10928299	A	T	A	T	-0.00036543	-0.000550461	0.532145	0.532153	2	147245005	0.0003	462933	0.071	463010	0.00010279	147245005	2	0.000379997
9	rs11022360	C	T	C	T	0.000416967	0.000980737	0.339112	0.339111	11	12479067	0.0003	462933	0.0024	463010	0.00010879	12479067	11	0.000129999
10	rs11059840	T	A	T	A	0.000456608	1.67182E-05	0.412168	0.412175	12	129189369	0.0003	462933	0.96	463010	0.00010408	129189369	12	1.09999E-05
11	rs11248859	A	G	A	G	-0.00045438	-3.59801E-05	0.376921	0.376932	16	1241997	0.0003	462933	0.91	463010	0.00010558	1241997	16	0.000017
12	rs11745114	C	T	C	T	-0.000483011	-0.000732277	0.557503	0.557503	5	174138977	0.0003	462933	0.018	463010	0.00010434	174138977	5	3.69999E-06
13	rs11765910	T	C	T	C	-0.000378577	0.000163543	0.440621	0.440623	7	131565550	0.0003	462933	0.6	463010	0.00010426	131565550	7	0.000280001
14	rs11976862	G	A	G	A	0.000420406	-2.90524E-05	0.453511	0.453501	7	8113312	0.0003	462933	0.92	463010	0.00010381	8113312	7	0.000051
15	rs1211547	C	T	C	T	-0.00037452	-0.000508449	0.474099	0.474095	12	128748579	0.0003	462933	0.099	463010	0.00010368	128748579	12	0.000299999
16	rs12246110	A	G	A	G	0.000395534	-5.70228E-05	0.351707	0.351706	10	128144886	0.0003	462933	0.86	463010	0.00010705	128144886	10	0.000219999
17	rs1225028	C	A	C	A	0.000423518	0.000354397	0.484821	0.484832	3	131659745	0.0003	462933	0.25	463010	0.00010269	131659745	3	3.69999E-05
18	rs12513847	T	C	T	C	0.000413004	0.000441898	0.502741	0.502741	5	125890559	0.0003	462933	0.15	463010	0.00010266	125890559	5	5.69994E-05
19	rs12545372	A	T	A	T	-0.00037694	-0.000185516	0.570396	0.570395	8	119822573	0.0003	462933	0.55	463010	0.00010478	119822573	8	0.00032
20	rs1259714	T	C	T	C	-0.000387479	-0.000186816	0.667791	0.667796	12	32018031	0.0003	462933	0.57	463010	0.00010951	32018031	12	0.0004
21	rs12910548	A	C	A	C	0.000397608	0.000679015	0.494744	0.494749	15	29056867	0.0003	462933	0.038	463010	0.00011043	29056867	15	0.00032
22	rs12910873	A	G	A	G	-0.000380808	0.000145158	0.398137	0.398144	15	71640074	0.0003	462933	0.64	463010	0.00010468	71640074	15	0.000280001
23	rs12921365	T	A	T	A	-0.00037096	-1.60552E-05	0.522919	0.52291	16	1225060	0.0003	462933	0.96	463010	0.00010471	1225060	16	0.0004
24	rs12954436	T	C	T	C	-0.000427456	-0.000184558	0.573152	0.573145	18	12436863	0.0003	462933	0.55	463010	0.00010392	12436863	18	3.89996E-05
25	rs13172608	T	C	T	C	-0.00041466	-0.000314676	0.393085	0.393085	5	25540446	0.0003	462933	0.31	463010	0.00010497	25540446	5	7.79992E-05
26	rs13400673	T	C	T	C	0.000424882	-0.000321205	0.441185	0.441192	2	123395921	0.0003	462933	0.33	463010	0.00011172	123395921	2	0.000140001
27	rs13414891	T	C	T	C	-0.000430458	-0.000117452	0.571268	0.571267	2	160915530	0.0003	462933	0.7	463010	0.00010339	160915530	2	3.09999E-05
28	rs13416767	G	A	G	A	0.0004026	0.000196088	0.674236	0.674237	2	106990423	0.0003	462933	0.55	463010	0.00011053	106990423	2	0.000269998
29	rs1347037	G	T	G	T	-0.000389093	-4.14687E-05	0.416682	0.416685	2	222057787	0.0003	462933	0.89	463010	0.00010388	222057787	2	0.000179999
30	rs1452091	G	T	G	T	-0.000403068	-0.00016972	0.31568	0.315675	21	28810976	0.0003	462933	0.61	463010	0.00011086	28810976	21	0.000280001
31	rs1472763	C	G	C	G	-0.000434087	-0.000481284	0.315773	0.315771	18	75846089	0.0003	462933	0.14	463010	0.00011071	75846089	18	8.79995E-05
32	rs1486785	T	C	T	C	-0.000398267	-0.000262817	0.534479	0.534481	4	30652381	0.0003	462933	0.39	463010	0.00010312	30652381	4	0.000109999
33	rs1573337	A	G	A	G	0.000396633	0.000554152	0.373204	0.373212	21	45249165	0.0003	462933	0.077	463010	0.00010571	45249165	21	0.000179999
34	rs1596456	A	T	A	T	0.00037223	0.000281002	0.397624	0.397618	3	59252776	0.0003	462933	0.37	463010	0.00010456	59252776	3	0.000369999
35	rs1657421	T	C	T	C	0.000375908	8.16845E-05	0.596703	0.596706	18	54658824	0.0003	462933	0.79	463010	0.00010444	54658824	18	0.00032
36	rs1673033	G	A	G	A	-0.000377502	-0.00023546	0.342098	0.342101	19	50940315	0.0003	462933	0.46	463010	0.00010786	50940315	19	0.000470002
37	rs17423839	G	A	G	A	0.000404241	0.000143196	0.436414	0.43642	7	32140526	0.0003	462933	0.64	463010	0.00010359	32140526	7	9.49992E-05
38	rs17485406	C	G	C	G	-0.000389911	-0.000392084	0.437497	0.4375	5	115356152	0.0003	462933	0.2	463010	0.00010324	115356152	5	0.00016
39	rs1961416	G	A	G	A	0.000413878	-0.000222825	0.638734	0.638745	2	179871172	0.0003	462933	0.48	463010	0.00010714	179871172	2	0.000109999
40	rs1977345	C	T	C	T	0.000364519	0.000202474	0.402768	0.402758	10	3276291	0.0003	462933	0.51	463010	0.00010443	3276291	10	0.000479999
41	rs1991483	G	A	G	A	-0.000374645	0.000455375	0.357805	0.357804	5	79039195	0.0003	462933	0.15	463010	0.00010715	79039195	5	0.000470002
42	rs2046420	T	G	T	G	0.000382968	0.000134944	0.587554	0.587556	2	151925287	0.0003	462933	0.66	463010	0.00010398	151925287	2	0.000230001
43	rs218178	A	G	A	G	0.000463075	8.43608E-05	0.550303	0.550301	2	33314920	0.0003	462933	0.78	463010	0.00010329	33314920	2	7.39997E-06
44	rs2243928	G	C	G	C	-0.000407772	-0.000603543	0.648933	0.648929	13	111981707	0.0003	462933	0.061	463010	0.00010849	111981707	13	0.00017
45	rs2244192	T	A	T	A	-0.000361341	-0.000516865	0.545363	0.545361	1	18890939	0.0003	462933	0.091	463010	0.00010285	18890939	1	0.000439997
46	rs2357154	T	A	T	A	0.000386075	9.62313E-05	0.579241	0.579243	2	193356300	0.0003	462933	0.75	463010	0.0001037	193356300	2	0.0002
47	rs242443	A	G	A	G	0.000367929	-0.000255213	0.496381	0.496383	20	58786958	0.0003	462933	0.41	463010	0.00010518	58786958	20	0.000470002
48	rs2424933	G	A	G	A	-0.000370456	-0.000494899	0.527786	0.527778	20	31636830	0.0003	462933	0.1	463010	0.00010242	31636830	20	0.000299999
49	rs2427460	C	T	C	T	0.000419377	0.000650764	0.476786	0.476777	20	61590782	0.0003	462933	0.034	463010	0.00010346	61590782	20	0.00005
50	rs2481961	T	C	T	C	-0.000449892	0.000192749	0.402997	0.402989	13	28531686	0.0003	462933	0.53	463010	0.00010436	28531686	13	0.000016
51	rs2486565	G	C	G	C	0.000381637	0.000675858	0.640005	0.640009	10	20804637	0.0003	462933	0.035	463010	0.00010774	20804637	10	0.0004
52	rs2619655	T	C	T	C	0.00035889	-2.09338E-05	0.536305	0.536303	10	68834808	0.0003	462933	0.95	463010	0.00010284	68834808	10	0.000479999
53	rs2647466	A	G	A	G	0.00041743	-0.000316074	0.370888	0.370885	1	220264588	0.0003	462933	0.32	463010	0.00010616	220264588	1	8.40001E-05
54	rs2664349	A	G	A	G	0.000370694	0.000530267	0.613109	0.613101	8	89089282	0.0003	462933	0.092	463010	0.00010588	89089282	8	0.000460002
55	rs2841415	G	A	G	A	-0.00044956	0.000224529	0.653247	0.653246	1	238926912	0.0003	462933	0.49	463010	0.00010931	238926912	1	3.89996E-05
56	rs28855701	G	A	G	A	-0.000402276	0.000221897	0.344226	0.34422	6	3761830	0.0003	462933	0.49	463010	0.00010862	3761830	6	0.00021
57	rs2911140	G	C	G	C	-0.000395235	-0.000723938	0.376582	0.376571	5	96179943	0.0003	462933	0.022	463010	0.00010618	96179943	5	0.0002
58	rs3123212	T	C	T	C	-0.000374131	-0.000175929	0.548911	0.548901	10	133189077	0.0003	462933	0.56	463010	0.00010291	133189077	10	0.000280001
59	rs3124596	A	G	A	G	-0.000373394	-0.000295914	0.485987	0.485993	9	139401504	0.0003	462933	0.33	463010	0.00010267	139401504	9	0.000280001
60	rs3127662	T	G	T	G	0.000403307	-0.000231465	0.444753	0.444745	6	107146996	0.0003	462933	0.45	463010	0.00010325	107146996	6	9.40005E-05
61	rs322403	A	G	A	G	0.000422834	0.000158917	0.414185	0.41418	4	187824275	0.0003	462933	0.61	463010	0.00010416	187824275	4	4.90004E-05
62	rs34569209	T	A	T	A	0.000382044	0.000349558	0.498816	0.498805	4	141022716	0.0003	462933	0.25	463010	0.00010265	141022716	4	0.0002
63	rs356313	A	G	A	G	0.000388745	0.000370201	0.343315	0.343319	4	68218680	0.0003	462933	0.25	463010	0.0001087	68218680	4	0.000350002
64	rs3774052	A	G	A	G	-0.000382614	-0.000294834	0.567136	0.567137	3	185884518	0.0003	462933	0.34	463010	0.00010426	185884518	3	0.000239999
65	rs4243461	A	G	A	G	0.000383866	0.000250667	0.648757	0.648759	6	139670131	0.0003	462933	0.43	463010	0.00010737	139670131	6	0.000350002
66	rs4296050	G	A	G	A	0.00038473	4.91999E-05	0.44405	0.444049	11	124386472	0.0003	462933	0.87	463010	0.00010416	124386472	11	0.000219999
67	rs4306623	C	T	C	T	0.000410956	0.000230426	0.621399	0.621406	18	69902385	0.0003	462933	0.47	463010	0.00010643	69902385	18	0.000109999
68	rs4457655	C	T	C	T	0.000387765	0.000356681	0.516001	0.516005	10	8926204	0.0003	462933	0.24	463010	0.00010222	8926204	10	0.00015
69	rs4671623	T	C	T	C	0.000383483	0.000434067	0.63666	0.636662	2	65126574	0.0003	462933	0.17	463010	0.00010663	65126574	2	0.00032
70	rs4689527	C	G	C	G	-0.000382713	0.000164024	0.636177	0.636167	4	6720977	0.0003	462933	0.61	463010	0.00010715	6720977	4	0.000350002
71	rs4736747	C	A	C	A	-0.000489949	0.000196179	0.451082	0.451078	8	132174914	0.0003	462933	0.52	463010	0.00010308	132174914	8	0.000002
72	rs4741368	T	A	T	A	0.000403732	-0.00030297	0.369088	0.36909	9	14376314	0.0003	462933	0.34	463010	0.00010772	14376314	9	0.000179999
73	rs4761637	C	T	C	T	-0.000420614	-0.000315012	0.580997	0.580998	12	94984559	0.0003	462933	0.31	463010	0.00010397	94984559	12	5.19996E-05
74	rs4770031	G	C	G	C	-0.000383549	0.000101683	0.663442	0.66343	13	21010688	0.0003	462933	0.75	463010	0.00010907	21010688	13	0.000439997
75	rs4802295	A	G	A	G	-0.00041298	-0.000151076	0.311532	0.311521	19	38733643	0.0003	462933	0.65	463010	0.00011104	38733643	19	0.0002
76	rs488808	C	A	C	A	0.00036039	0.000344269	0.538051	0.538033	11	131731244	0.0003	462933	0.26	463010	0.00010339	131731244	11	0.000490004
77	rs4908532	T	C	T	C	0.000374594	0.000352546	0.616405	0.616421	1	9389370	0.0003	462933	0.26	463010	0.00010539	9389370	1	0.000379997
78	rs55687407	C	A	C	A	0.000383249	0.000173348	0.516039	0.516023	4	18185371	0.0003	462933	0.57	463010	0.00010268	18185371	4	0.000189998
79	rs56249560	T	C	T	C	0.00039097	-0.00038845	0.38388	0.38388	2	175607716	0.0003	462933	0.22	463010	0.00010559	175607716	2	0.00021
80	rs584976	T	C	T	C	-0.000403304	-0.000337662	0.358233	0.358227	1	186193218	0.0003	462933	0.29	463010	0.00010682	186193218	1	0.00016
81	rs6035872	A	G	A	G	-0.000393605	-0.000477199	0.618624	0.618619	20	2196285	0.0003	462933	0.13	463010	0.00010555	2196285	20	0.000189998
82	rs606724	G	A	G	A	-0.000381499	-0.000295296	0.382048	0.382051	3	43100057	0.0003	462933	0.35	463010	0.00010553	43100057	3	0.000299999
83	rs62488405	A	C	A	C	0.00042163	0.000697318	0.357547	0.357556	7	86234000	0.0003	462933	0.028	463010	0.00010695	86234000	7	8.09991E-05
84	rs6494040	T	C	T	C	-0.000389487	0.000304638	0.671972	0.671973	15	32193644	0.0003	462933	0.35	463010	0.00010911	32193644	15	0.000359998

85	rs6558394	A	G	A	G	-0.000376181	-0.0004128	0.643869	0.643875	8	144893084	0.0003	462933	0.19	463010	0.00010688	144893084	8	0.000430002
86	rs6561273	G	C	G	C	0.000433149	0.00044896	0.386166	0.386166	13	46514492	0.0003	462933	0.15	463010	0.00010595	46514492	13	4.30002E-05
87	rs6665227	C	T	C	T	0.000407217	0.000599406	0.356872	0.356876	1	155710460	0.0003	462933	0.059	463010	0.00010696	155710460	1	0.000140001
88	rs66867810	A	G	A	G	-0.000402395	-0.000715264	0.324115	0.324105	6	41940886	0.0003	462933	0.028	463010	0.00010944	41940886	6	0.000239999
89	rs6707444	C	T	C	T	-0.000403507	6.75727E-05	0.590257	0.590247	2	231225509	0.0003	462933	0.83	463010	0.0001043	231225509	2	0.000109999
90	rs67396441	T	C	T	C	0.000417551	0.000152299	0.363458	0.363469	2	127772373	0.0003	462933	0.64	463010	0.00010919	127772373	2	0.000129999
91	rs6772866	T	G	T	G	0.000374712	0.000212542	0.399798	0.399805	3	151672788	0.0003	462933	0.5	463010	0.00010621	151672788	3	0.000420001
92	rs6779574	A	G	A	G	0.00038524	-0.000476407	0.335201	0.3352	3	126003796	0.0003	462933	0.14	463010	0.00010829	126003796	3	0.000369999
93	rs6783259	C	T	C	T	0.000392164	-0.000407974	0.569238	0.569243	3	63219932	0.0003	462933	0.19	463010	0.00010391	63219932	3	0.00016
94	rs6889925	A	G	A	G	0.00038212	6.35108E-05	0.406255	0.406251	5	73579842	0.0003	462933	0.84	463010	0.00010362	73579842	5	0.000230001
95	rs6909005	A	G	A	G	0.000372947	0.000160283	0.582092	0.582095	6	68954012	0.0003	462933	0.6	463010	0.00010371	68954012	6	0.00032
96	rs7095505	G	C	G	C	-0.000390346	0.000334202	0.64436	0.644363	10	73686961	0.0003	462933	0.29	463010	0.00010734	73686961	10	0.000280001
97	rs7169551	G	A	G	A	0.000401172	0.000337421	0.419874	0.41986	15	95303075	0.0003	462933	0.27	463010	0.00010402	95303075	15	0.000109999
98	rs7193317	G	C	G	C	-0.000373516	0.000136336	0.640806	0.640805	16	19502802	0.0003	462933	0.67	463010	0.00010665	19502802	16	0.000460002
99	rs7298366	C	T	C	T	-0.000388193	-0.000395862	0.430233	0.43023	12	22980293	0.0003	462933	0.2	463010	0.00010364	22980293	12	0.000179999
100	rs73169193	T	C	T	C	-0.000376092	0.000124599	0.363621	0.363617	22	42751397	0.0003	462933	0.7	463010	0.00010736	42751397	22	0.000460002
101	rs7775754	C	G	C	G	-0.000388417	0.000461721	0.630044	0.630049	6	120309995	0.0003	462933	0.14	463010	0.00010608	120309995	6	0.00025
102	rs7850966	G	C	G	C	0.000450995	0.000478376	0.659693	0.659703	9	19125528	0.0003	462933	0.14	463010	0.00010847	19125528	9	0.000032
103	rs7911581	A	C	A	C	0.000428483	0.000124466	0.3358	0.335793	10	14169002	0.0003	462933	0.7	463010	0.00010865	14169002	10	0.00008
104	rs8069046	C	T	C	T	0.000435109	0.000142174	0.489225	0.489212	17	80804183	0.0003	462933	0.67	463010	0.00011094	80804183	17	8.79995E-05
105	rs822320	T	G	T	G	-0.000402693	-0.000189329	0.382993	0.382995	8	16439914	0.0003	462933	0.55	463010	0.00010536	16439914	8	0.000129999
106	rs946812	G	C	G	C	0.000381218	0.000467257	0.525853	0.525846	10	16201323	0.0003	462933	0.12	463010	0.00010247	16201323	10	0.0002
107	rs9513601	A	G	A	G	-0.000373423	0.000114679	0.489475	0.489471	13	100000134	0.0003	462933	0.71	463010	0.0001024	100000134	13	0.000269998
108	rs9545611	C	T	C	T	0.000388694	0.000355755	0.640938	0.64094	13	81759788	0.0003	462933	0.27	463010	0.00010819	81759788	13	0.000329997
109	rs9577288	C	T	C	T	-0.000401149	-0.0002532	0.425568	0.425568	13	112530889	0.0003	462933	0.41	463010	0.00010401	112530889	13	0.000109999
110	rs9628245	C	G	C	G	-0.000428102	-0.000884022	0.380135	0.38013	22	51174048	0.0003	462933	0.011	463010	0.00011671	51174048	22	0.000239999
111	rs9812247	G	A	G	A	-0.000446426	-4.74952E-05	0.339585	0.339571	3	1918651	0.0003	462933	0.88	463010	0.00011047	1918651	3	5.30005E-05

ukb-a-55 SNPs

	SNP	effect allele.exposure	other allele.exposure	effect allele.outcome	other allele.outcome	beta.exposure	beta.outcome	eaf.exposure	eaf.outcome	chr	pos	se.outcon	samplesi	pval.outc	samplesi	se.exposure	pos.exposure	chr.expos	pval.exposure
1	rs10263370	T	C	T	C	-0.000370343	-9.52219E-05	0.462431	0.461457	7	75413948	0.0004	337159	0.7934	463010	0.00010478	75413948	7	0.000409996
2	rs10400592	A	G	A	G	0.000408049	0.000328687	0.345315	0.345857	12	73131071	0.0004	337159	0.3791	463010	0.00010769	73131071	12	0.00015
3	rs10736856	T	C	T	C	0.000385383	-0.000178378	0.679632	0.678841	9	135490787	0.0004	337159	0.6393	463010	0.00010969	135490787	9	0.000439997
4	rs10739224	G	T	G	T	0.000429701	0.000118642	0.376959	0.374049	9	109450946	0.0004	337159	0.7486	463010	0.00010641	109450946	9	5.39995E-05
5	rs10747846	G	C	G	C	0.000389305	3.90915E-05	0.511668	0.514093	12	59928206	0.0004	337159	0.9127	463010	0.00010263	59928206	12	0.00015
6	rs10848951	C	T	C	T	0.000431645	-0.000139391	0.397641	0.398858	12	3996234	0.0004	337159	0.7026	463010	0.00010517	3996234	12	4.09996E-05
7	rs10867416	C	T	C	T	-0.00043174	0.000013425	0.655657	0.655622	9	82324152	0.0004	337159	0.9714	463010	0.0001079	82324152	9	6.29999E-05
8	rs10928299	A	T	A	T	-0.00036543	-0.000558154	0.532145	0.53329	2	147245005	0.0004	337159	0.118	463010	0.00010279	147245005	2	0.000379997
9	rs11022360	C	T	C	T	0.000416967	0.00133727	0.339112	0.337717	11	12479067	0.0004	337159	0.0004	463010	0.00010879	12479067	11	0.000129999
10	rs11059840	T	A	T	A	0.000456608	-0.000209265	0.412168	0.413592	12	129189369	0.0004	337159	0.5625	463010	0.00010408	129189369	12	1.09999E-05
11	rs11248859	A	G	A	G	-0.00045438	-4.51172E-05	0.376921	0.379923	16	1241997	0.0004	337159	0.902	463010	0.00010558	1241997	16	0.000017
12	rs11745114	C	T	C	T	-0.000483011	-0.000677323	0.557503	0.561046	5	174138977	0.0004	337159	0.0617	463010	0.00010434	174138977	5	3.69999E-06
13	rs11765910	T	C	T	C	-0.000378577	8.94235E-05	0.440621	0.444286	7	131565550	0.0004	337159	0.8049	463010	0.00010426	131565550	7	0.000280001
14	rs11976862	G	A	G	A	0.000420406	-4.76265E-05	0.453511	0.450526	7	8113312	0.0004	337159	0.895	463010	0.00010381	8113312	7	0.000051
15	rs1211547	C	T	C	T	-0.00037452	-0.000733718	0.474099	0.471843	12	128748579	0.0004	337159	0.0416	463010	0.00010368	128748579	12	0.000299999
16	rs12246110	A	G	A	G	0.000395534	-6.50968E-05	0.351707	0.352664	10	128144886	0.0004	337159	0.8609	463010	0.00010705	128144886	10	0.000219999
17	rs1225028	C	A	C	A	0.000423518	0.000580131	0.484821	0.485982	3	131659745	0.0004	337159	0.1035	463010	0.00010269	131659745	3	3.69999E-05
18	rs12513847	T	C	T	C	0.000413004	0.000335678	0.502741	0.503058	5	125890559	0.0004	337159	0.3466	463010	0.00010266	125890559	5	5.69994E-05
19	rs12545372	A	T	A	T	-0.00037694	-0.000345798	0.570396	0.574898	8	119822573	0.0004	337159	0.3429	463010	0.00010478	119822573	8	0.00032
20	rs1259714	T	C	T	C	-0.000387479	3.34793E-05	0.667791	0.665613	12	32018031	0.0004	337159	0.9298	463010	0.00010951	32018031	12	0.0004
21	rs12910548	A	C	A	C	0.000397608	0.000608727	0.494744	0.491488	15	29056867	0.0004	337159	0.1125	463010	0.00011043	29056867	15	0.00032
22	rs12910873	A	G	A	G	-0.000380808	0.000419875	0.398137	0.399286	15	71640074	0.0004	337159	0.2478	463010	0.00010468	71640074	15	0.000280001
23	rs12921365	T	A	T	A	-0.00037096	-9.09031E-05	0.522919	0.524	16	1225060	0.0004	337159	0.8025	463010	0.00010471	1225060	16	0.0004
24	rs12954436	T	C	T	C	-0.000427456	-0.000408955	0.573152	0.572374	18	12436863	0.0004	337159	0.2571	463010	0.00010392	12436863	18	3.89996E-05
25	rs13172608	T	C	T	C	-0.00041466	-0.000110129	0.393085	0.392311	5	25540446	0.0004	337159	0.7627	463010	0.00010497	25540446	5	7.79992E-05
26	rs13400673	T	C	T	C	0.000424882	3.02736E-05	0.441185	0.443132	2	123395921	0.0004	337159	0.9378	463010	0.00011172	123395921	2	0.000140001
27	rs13414891	T	C	T	C	-0.000430458	-0.000223739	0.571268	0.576216	2	160915530	0.0004	337159	0.5344	463010	0.00010339	160915530	2	3.09999E-05
28	rs13416767	G	A	G	A	0.0004026	3.57875E-06	0.674236	0.676134	2	106990423	0.0004	337159	0.9926	463010	0.00011053	106990423	2	0.000269998
29	rs1347037	G	T	G	T	-0.000389093	-0.000416059	0.416682	0.412476	2	222057787	0.0004	337159	0.2499	463010	0.00010388	222057787	2	0.000179999
30	rs1452091	G	T	G	T	-0.000403068	-0.000124633	0.31568	0.314338	21	28810976	0.0004	337159	0.7463	463010	0.00011086	28810976	21	0.000280001
31	rs1472763	C	G	C	G	-0.000434087	-0.000732986	0.315773	0.315493	18	75846089	0.0004	337159	0.0565	463010	0.00011071	75846089	18	8.79995E-05
32	rs1486785	T	C	T	C	-0.000398267	-0.000302696	0.534479	0.537121	4	30652381	0.0004	337159	0.3986	463010	0.00010312	30652381	4	0.000109999
33	rs1573337	A	G	A	G	0.000396633	0.000350763	0.373204	0.370904	21	45249165	0.0004	337159	0.34	463010	0.00010571	45249165	21	0.000179999
34	rs1596456	A	T	A	T	0.00037223	-9.70953E-05	0.397624	0.397436	3	59252776	0.0004	337159	0.7893	463010	0.00010456	59252776	3	0.000369999
35	rs1657421	T	C	T	C	0.000375908	-6.30889E-06	0.596703	0.596106	18	54658824	0.0004	337159	0.9861	463010	0.00010444	54658824	18	0.00032
36	rs1673033	G	A	G	A	-0.000377502	0.000136412	0.342098	0.339885	19	50940315	0.0004	337159	0.7164	463010	0.00010786	50940315	19	0.000470002
37	rs17423839	G	A	G	A	0.000404241	0.00064247	0.436414	0.43864	7	32140526	0.0004	337159	0.0739	463010	0.00010359	32140526	7	9.49992E-05
38	rs17485406	C	G	C	G	-0.000389911	-0.000449051	0.437497	0.436156	5	115356152	0.0004	337159	0.2104	463010	0.00010324	115356152	5	0.00016
39	rs1961416	G	A	G	A	0.000413878	-0.000414956	0.638734	0.640819	2	179871172	0.0004	337159	0.2656	463010	0.00010714	179871172	2	0.000109999
40	rs1977345	C	T	C	T	0.000364519	0.000486004	0.402768	0.402952	10	3276291	0.0004	337159	0.1803	463010	0.00010443	3276291	10	0.000479999
41	rs1991483	G	A	G	A	-0.000374645	0.00019632	0.357805	0.355038	5	79039195	0.0004	337159	0.5981	463010	0.00010715	79039195	5	0.000470002
42	rs2046420	T	G	T	G	0.000382968	-0.000100713	0.587554	0.58924	2	151925287	0.0004	337159	0.7805	463010	0.00010398	151925287	2	0.000230001
43	rs218178	A	G	A	G	0.000463075	-9.74778E-05	0.550303	0.548646	2	33314920	0.0004	337159	0.7858	463010	0.00010329	33314920	2	7.39997E-06
44	rs2243928	G	C	G	C	-0.000407772	-0.000606513	0.648933	0.647078	13	111981707	0.0004	337159	0.1072	463010	0.00010849	111981707	13	0.00017
45	rs2244192	T	A	T	A	-0.000361341	-0.000425329	0.545363	0.546293	1	18890939	0.0004	337159	0.2339	463010	0.00010285	18890939	1	0.000439997
46	rs2357154	T	A	T	A	0.000386075	-0.000152115	0.579241	0.58084	2	193356300	0.0004	337159	0.6731	463010	0.0001037	193356300	2	0.0002
47	rs242443	A	G	A	G	0.000367929	0.000165153	0.496381	0.500992	20	58786958	0.0004	337159	0.6513	463010	0.00010518	58786958	20	0.000470002
48	rs2424933	G	A	G	A	-0.000370456	-0.000818907	0.527786	0.528965	20	31636830	0.0004	337159	0.0213	463010	0.00010242	31636830	20	0.000299999
49	rs2427460	C	T	C	T	0.000419377	0.000381376	0.476786	0.474214	20	61590782	0.0004	337159	0.2887	463010	0.00010346	61590782	20	0.00005
50	rs2481961	T	C	T	C	-0.000449892	0.00018111	0.402997	0.399958	13	28531686	0.0004	337159	0.6181	463010	0.00010436	28531686	13	0.000016
51	rs2486565	G	C	G	C	0.000381637	0.000927646	0.640005	0.639078	10	20804637	0.0004	337159	0.0132	463010	0.00010774	20804637	10	0.0004
52	rs2619655	T	C	T	C	0.00035889	0.000254629	0.536305	0.533418	10	68834808	0.0004	337159	0.4761	463010	0.00010284	68834808	10	0.000479999
53	rs2647466	A	G	A	G	0.00041743	-0.000621997	0.370888	0.368216	1	220264588	0.0004	337159	0.0924	463010	0.00010616	220264588	1	8.40001E-05
54	rs2664349	A	G	A	G	0.000370694	0.000454943	0.613109	0.611338	8	89089282	0.0004	337159	0.2154	463010	0.00010588	89089282	8	0.000460002
55	rs2841415	G	A	G	A	-0.00044956	0.000371853	0.653247	0.652035	1	238926912	0.0004	337159	0.3268	463010	0.00010931	238926912	1	3.89996E-05
56	rs28855701	G	A	G	A	-0.000402276	0.000279107	0.344226	0.346785	6	3761830	0.0004	337159	0.4595	463010	0.00010862	3761830	6	0.00021
57	rs2911140	G	C	G	C	-0.000395235	-0.00086584	0.376582	0.377659	5	96179943	0.0004	337159	0.0189	463010	0.00010618	96179943	5	0.0002
58	rs3123212	T	C	T	C	-0.000374131	-0.000349011	0.548911	0.5532	10	133189077	0.0004	337159	0.3299	463010	0.00010291	133189077	10	0.000280001
59	rs3124596	A	G	A	G	-0.000373394	-0.000120248	0.485987	0.482174	9	139401504	0.0004	337159	0.7361	463010	0.00010267	139401504	9	0.000280001
60	rs3127662	T	G	T	G	0.000403307	-0.00012429	0.444753	0.447337	6	107146996	0.0004	337159	0.7288	463010	0.00010325	107146996	6	9.40005E-05
61	rs322403	A	G	A	G	0.000422834	-4.68748E-05	0.414185	0.413765	4	187824275	0.0004	337159	0.8969	463010	0.00010416	187824275	4	4.90004E-05
62	rs34569209	T	A	T	A	0.000382044	-0.000396536	0.498816	0.499316	4	141022716	0.0004	337159	0.2663	463010	0.00010265	141022716	4	0.0002
63	rs356313	A	G	A	G	0.000388745	0.000438694	0.343315	0.342179	4	68218680	0.0004	337159	0.2455	463010	0.0001087	68218680	4	0.000350002
64	rs3774052	A	G	A	G	-0.000382614	-0.000291919	0.567136	0.566219	3	185884518	0.0004	337159	0.4201	463010	0.00010426	185884518	3	0.000239999
65	rs4243461	A	G	A	G	0.000383866	0.000236093	0.648757	0.649218	6	139670131	0.0004	337159	0.527	463010	0.00010737	139670131	6	0.000350002
66	rs4296050	G	A	G	A	0.00038473	3.42758E-05	0.44405	0.442506	11	124386472	0.0004	337159	0.9245	463010	0.00010416	124386472	11	0.000219999
67	rs4306623	C	T	C	T	0.000410956	6.39507E-05	0.621399	0.620946	18	69902385	0.0004	337159	0.8627	463010	0.00010643	69902385	18	0.000109999
68	rs4457655	C	T	C	T	0.000387765	0.000437402	0.516001	0.516001	10	8926204	0.0004	337159	0.2184	463010	0.00010222	8926204	10	0.00015
69	rs4671623	T	C	T	C	0.000383483	0.000446847	0.63666	0.638077	2	65126574	0.0004	337159	0.2282	463010	0.00010663	65126574	2	0.00032
70	rs4689527	C	G	C	G	-0.000382713	-0.000242115	0.636177	0.640236	4	6720977	0.0004	337159	0.5158	463010	0.00010715	6720977	4	0.000350002
71	rs4736747	C	A	C	A	-0.000489949	1.58235E-05	0.451082	0.448922	8	132174914	0.0004	337159	0.9648	463010	0.00010308	132174914	8	0.000002
72	rs4741368	T	A	T	A	0.000403732	0.000079699	0.369088	0.369662	9	14376314	0.0004	337159	0.8312	463010	0.00010772	14376314	9	0.000179999
73	rs4761637	C	T	C	T	-0.000420614	1.40372E-05	0.580997	0.582004	12	94984559	0.0004	337159	0.969	463010	0.00010397	94984559	12	5.19996E-05
74	rs4770031	G	C	G	C	-0.000383549	-6.21304E-05	0.663442	0.662068	13	21010688	0.0004	337159	0.8696	463010	0.00010907	21010688	13	0.000439997
75	rs4802295	A	G	A	G	-0.00041298	-0.000002356	0.311532	0.311004	19	38733643	0.0004	337159	0.9951	463010	0.00011104	38733643	19	0.0002
76	rs488808	C	A	C	A	0.00036039	0.000318904	0.538051	0.537889	11	131731244	0.0004	337159	0.3744	463010	0.00010339	131731244	11	0.000490004
77	rs4908532	T	C	T	C	0.000374594	0.0004082	0.616405	0.617609	1	9389370	0.0004	337159	0.2651	463010	0.00010539	9389370	1	0.000379997
78	rs55687407	C	A	C	A	0.000383249	0.000094213	0.516039	0.514717	4	18185371	0.0004	337159	0.7915	463010	0.00010268	18185371	4	0.000189998
79	rs56249560	T	C	T	C	0.00039097	-0.000421503	0.38388	0.385667	2	175607716	0.0004	337159	0.2498	463010	0.00010559	175607716	2	0.00021
80	rs584976	T	C	T	C	-0.000403304	3.96898E-05	0.358233	0.360659	1	186193218	0.0004	337159	0.9147	463010	0.00010682	186193218	1	0.00016
81	rs6035872	A	G	A	G	-0.000393605	-0.000516145	0.618624	0.618716	20	2196285	0.0004	337159	0.1594	463010	0.00010555	2196285	20	0.000189998
82	rs606724	G	A	G	A	-0.000381499	-0.000230545	0.382048	0.382555	3	43100057	0.0004	337159	0.5294	463010	0.00010553	43100057	3	0.000299999
83	rs62488405	A	C	A	C	0.00042163	0.000325355	0.357547	0.359857	7	86234000	0.0004	337159	0.3808	463010	0.00010695	86234000	7	8.09991E-05
84	rs6494040	T	C	T	C	-0.000389487	0.000641279	0.671972	0.673263	15	32193644	0.0004	337159	0.0912	463010	0.00010911	32193644	15	0.000359998

85	rs6558394	A	G	A	G	-0.000376181	-0.00052218	0.643869	0.647251	8	144893084	0.0004	337159	0.1605	463010	0.00010688	144893084	8	0.000430002
86	rs6561273	G	C	G	C	0.000433149	0.00058284	0.386166	0.38532	13	46514492	0.0004	337159	0.1129	463010	0.00010595	46514492	13	4.30002E-05
87	rs6665227	C	T	C	T	0.000407217	0.000424831	0.356872	0.358763	1	155710460	0.0004	337159	0.2525	463010	0.00010696	155710460	1	0.000140001
88	rs66867810	A	G	A	G	-0.000402395	-0.000593479	0.324115	0.322114	6	41940886	0.0004	337159	0.1191	463010	0.00010944	41940886	6	0.000239999
89	rs6707444	C	T	C	T	-0.000403507	0.000132958	0.590257	0.586999	2	231225509	0.0004	337159	0.7136	463010	0.0001043	231225509	2	0.000109999
90	rs67396441	T	C	T	C	0.000417551	0.000185945	0.363458	0.36372	2	127772373	0.0004	337159	0.6234	463010	0.00010919	127772373	2	0.000129999
91	rs6772866	T	G	T	G	0.000374712	0.000359954	0.399798	0.401461	3	151672788	0.0004	337159	0.329	463010	0.00010621	151672788	3	0.000420001
92	rs6779574	A	G	A	G	0.00038524	-0.000618023	0.335201	0.334716	3	126003796	0.0004	337159	0.1004	463010	0.00010829	126003796	3	0.000369999
93	rs6783259	C	T	C	T	0.000392164	-0.000181394	0.569238	0.568132	3	63219932	0.0004	337159	0.6153	463010	0.00010391	63219932	3	0.00016
94	rs6889925	A	G	A	G	0.00038212	0.000108693	0.406255	0.405793	5	73579842	0.0004	337159	0.7628	463010	0.00010362	73579842	5	0.000230001
95	rs6909005	A	G	A	G	0.000372947	-3.09106E-05	0.582092	0.581473	6	68954012	0.0004	337159	0.9316	463010	0.00010371	68954012	6	0.00032
96	rs7095505	G	C	G	C	-0.000390346	0.000681368	0.64436	0.646833	10	73686961	0.0004	337159	0.0682	463010	0.00010734	73686961	10	0.000280001
97	rs7169551	G	A	G	A	0.000401172	0.000325309	0.419874	0.416822	15	95303075	0.0004	337159	0.3684	463010	0.00010402	95303075	15	0.000109999
98	rs7193317	G	C	G	C	-0.000373516	0.000307273	0.640806	0.64258	16	19502802	0.0004	337159	0.4075	463010	0.00010665	19502802	16	0.000460002
99	rs7298366	C	T	C	T	-0.000388193	-0.000201595	0.430233	0.431744	12	22980293	0.0004	337159	0.5752	463010	0.00010364	22980293	12	0.000179999
100	rs73169193	T	C	T	C	-0.000376092	0.000317015	0.363621	0.365055	22	42751397	0.0004	337159	0.3948	463010	0.00010736	42751397	22	0.000460002
101	rs7775754	C	G	C	G	-0.000388417	0.000505714	0.630044	0.630429	6	120309995	0.0004	337159	0.17	463010	0.00010608	120309995	6	0.00025
102	rs7850966	G	C	G	C	0.000450995	0.000471115	0.659693	0.662401	9	19125528	0.0004	337159	0.2119	463010	0.00010847	19125528	9	0.000032
103	rs7911581	A	C	A	C	0.000428483	0.000535494	0.3358	0.33552	10	14169002	0.0004	337159	0.1557	463010	0.00010865	14169002	10	0.00008
104	rs8069046	C	T	C	T	0.000435109	0.000671073	0.489225	0.514692	17	80804183	0.0004	337159	0.0813	463010	0.00011094	80804183	17	8.79995E-05
105	rs822320	T	G	T	G	-0.000402693	-0.000294994	0.382993	0.382982	8	16439914	0.0004	337159	0.4203	463010	0.00010536	16439914	8	0.000129999
106	rs946812	G	C	G	C	0.000381218	0.000369191	0.525853	0.523614	10	16201323	0.0004	337159	0.2995	463010	0.00010247	16201323	10	0.0002
107	rs9513601	A	G	A	G	-0.000373423	-8.10908E-05	0.489475	0.491701	13	100000134	0.0004	337159	0.8198	463010	0.0001024	100000134	13	0.000269998
108	rs9545611	C	T	C	T	0.000388694	0.00030362	0.640938	0.643123	13	81759788	0.0004	337159	0.4202	463010	0.00010819	81759788	13	0.000329997
109	rs9577288	C	T	C	T	-0.000401149	-0.000179747	0.425568	0.424353	13	112530889	0.0004	337159	0.6188	463010	0.00010401	112530889	13	0.000109999
110	rs9628245	C	G	C	G	-0.000428102	-0.000976952	0.380135	0.358178	22	51174048	0.0004	337159	0.0157	463010	0.00011671	51174048	22	0.000239999
111	rs9812247	G	A	G	A	-0.000446426	-0.000132625	0.339585	0.338013	3	1918651	0.0004	337159	0.7296	463010	0.00011047	1918651	3	5.30005E-05
